# Clinical course and predictors of 60-day mortality in 239 critically ill patients with COVID-19: a multicenter retrospective study from Wuhan, China

**DOI:** 10.1186/s13054-020-03098-9

**Published:** 2020-07-06

**Authors:** Jiqian Xu, Xiaobo Yang, Luyu Yang, Xiaojing Zou, Yaxin Wang, Yongran Wu, Ting Zhou, Yin Yuan, Hong Qi, Shouzhi Fu, Hong Liu, Jia’an Xia, Zhengqin Xu, Yuan Yu, Ruiting Li, Yaqi Ouyang, Rui Wang, Lehao Ren, Yingying Hu, Dan Xu, Xin Zhao, Shiying Yuan, Dingyu Zhang, You Shang

**Affiliations:** 1 Department of Critical Care Medicine, Union Hospital, Tongji Medical College, Huazhong University of Science and Technology, Wuhan, China; 2 Research Center for Translational Medicine, Jinyintan Hospital, Wuhan, China; 3 Department of ICU/Emergency Wuhan Third Hospital, Wuhan University, Wuhan, China; 4 Department of Critical Care Medicine, Xiangyang No.1 Hospital, Affiliated Hospital of Hubei University of Medicine, Xiangyang, China

**Keywords:** COVID-19, Mortality, Thrombocytopenia, Acute respiratory syndrome, Acute kidney injury

## Abstract

**Background:**

The global numbers of confirmed cases and deceased critically ill patients with COVID-19 are increasing. However, the clinical course, and the 60-day mortality and its predictors in critically ill patients have not been fully elucidated. The aim of this study is to identify the clinical course, and 60-day mortality and its predictors in critically ill patients with COVID-19.

**Methods:**

Critically ill adult patients admitted to intensive care units (ICUs) from 3 hospitals in Wuhan, China, were included. Data on demographic information, preexisting comorbidities, laboratory findings at ICU admission, treatments, clinical outcomes, and results of SARS-CoV-2 RNA tests and of serum SARS-CoV-2 IgM were collected including the duration between symptom onset and negative conversion of SARS-CoV-2 RNA.

**Results:**

Of 1748 patients with COVID-19, 239 (13.7%) critically ill patients were included. Complications included acute respiratory distress syndrome (ARDS) in 164 (68.6%) patients, coagulopathy in 150 (62.7%) patients, acute cardiac injury in 103 (43.1%) patients, and acute kidney injury (AKI) in 119 (49.8%) patients, which occurred 15.5 days, 17 days, 18.5 days, and 19 days after the symptom onset, respectively. The median duration of the negative conversion of SARS-CoV-2 RNA was 30 (range 6–81) days in 49 critically ill survivors that were identified. A total of 147 (61.5%) patients deceased by 60 days after ICU admission. The median duration between ICU admission and decease was 12 (range 3–36). Cox proportional-hazards regression analysis revealed that age older than 65 years, thrombocytopenia at ICU admission, ARDS, and AKI independently predicted the 60-day mortality.

**Conclusions:**

Severe complications are common and the 60-day mortality of critically ill patients with COVID-19 is considerably high. The duration of the negative conversion of SARS-CoV-2 RNA and its association with the severity of critically ill patients with COVID-19 should be seriously considered and further studied.

## Background

The epidemic of novel coronavirus disease 2019 (COVID-19) struck Wuhan in late December 2019. As of June 8, 2020, the global number of confirmed cases has reached 6,931,000, with 400,857 deaths [1]. Although most patients recovered from COVID-19, critically ill patients need long-term hospitalizations and have a considerable risk of death. Our previous small sample-sized study showed that approximately 7% of inpatients with COVID-19 developed critical complications. Among the critically ill patients, by 28-day after admission to an intensive care unit (ICU), 61.5% deceased, and 60% of the survivors were still in hospital [2]. The long-term prognosis of all survivors is unknown.

Critically ill patients with COVID-19 are characterized by progressive respiratory failure due to lung infection of severe acute respiratory syndrome coronavirus 2 (SARS-CoV-2) [3]. Recent evidence suggested that SARS-CoV-2 might directly dysregulate the normal functions of the kidney, liver, and peripheral blood components, which increases the risk of multiple organ failure [3–5]. To our knowledge, some potential high-risk factors of death were speculated in small sample-sized studies on COVID-19 [2, 4, 6]. In another study exploring the risk factors of death, only 11 (8%) were treated in ICUs and only 3 (2%) received mechanical ventilation during hospitalization in the survivor group, and severe complications during the progression of COVID-19, including acute respiratory syndrome (ARDS), acute cardiac injury, acute kidney injury (AKI), and liver dysfunction, were not considered at all [7].

Here, for this WHO-declared pandemic [8], we intended to report our findings on clinical course and the 60-day mortality in 239 critically ill patients with COVID-19 from Wuhan, China.

## Methods

### Study design and participants

We aimed to retrospectively study critically ill adult patients with COVID-19 admitted into ICUs from Wuhan Union Hospital, Jinyintan Hospital, and Wuhan Third Hospital, from January 12 to February 3, 2020. As previously described, SARS-CoV-2 infections were confirmed by a positive result on a reverse transcriptase-polymerase chain reaction (RT-PCR) assay of specimens from the respiratory tract according to guidelines released by National Health Commission of the People’s Republic of China [9]. Critically ill patients were defined to be individuals admitted to ICU, who required mechanical ventilation or had a fraction of inspired oxygen (FiO_2_) concentration greater than or equal to 60% as described in previous reports [2, 8, 10, 11]. Critically ill patients who deceased within 48 h after ICU admission were excluded, because their durations in ICUs were too short to reveal the effectiveness of treatments received in ICUs and to eliminate the bias on data collection of organ function or complications. Research approval (KY-2020-23.01) was granted by the ethics board of Jin Yin-tan Hospital as the central coordinating center. The need for informed consent was waived.

### Criteria for ICU admission and treatment

The ICU admission criteria and treatment decisions for all patients, including determination of the need for intubation and respiratory support, were made at the discretion of the treating physicians and were not standardized. In general, the goal is to ascertain adequate oxygenation to maintain SpO_2_ ≥ 90% through high-flow nasal cannula (HFNC) and noninvasive ventilation (NIV) [12–14]. If the respiratory failure progressively deteriorated, the patients were considered to be eligible for noninvasive or invasive mechanical ventilation when PaO_2_/FiO_2_ ≤ 200 mmHg. Where available, in patients with refractory hypoxemia (PaO_2_/FiO_2_ < 80 or 60 mmHg) veno-venous extracorporeal membrane oxygenation (ECMO) might serve as a therapeutic option to stabilize gas exchange [13, 14].

### Data collection

Patient identification in the three hospitals was achieved by reviewing admission logs from available medical records. After several cycles of feedback and pilot testing, modified case report forms referencing the case record form shared by the International Severe Acute Respiratory and Emerging Infection Consortium for SARS-CoV-2 infection. Data were extracted from local servers by experienced research physicians at each center.

Demographic data, preexisting comorbidities, vital signs at ICU admission, laboratory values at ICU admission, complications, treatments, and test result of SARS-CoV-2 RNA on samples from the respiratory tract and of serum SARS-CoV-2 IgM were collected. For patients discharged, phone calls were made by April 5, 2020, to record their living status.

### Outcomes and definitions

The primary outcome was 60-day mortality and its predictors. ARDS were defined according to the Berlin Definition [15]. AKI was diagnosed according to KDIGO clinical practice guidelines based on the serum creatinine levels [16]. Acute cardiac injury was diagnosed if the serum concentration of hsTNI was measured in the laboratory above the upper limit of the reference range (> 28 pg/mL) [2]. Liver dysfunction was diagnosed if serum ALT > 50 U/L or AST > 40 U/L during disease progression [5]. Coagulopathy was defined if PT > 13.5 s or APTT > 37 s. Negative conversion of SARS-CoV-2 RNA was defined as the last time when SARS-CoV-2 RNA was tested positive on samples from the respiratory tract. Hospital-acquired infection was diagnosed if the patients had a positive culture of a new pathogen obtained from lower respiratory tract specimens (bacterial pneumonia), blood samples (bacteremia), or urine (urinary tract infection) taken ≥ 48 h after ICU admission [17].

### Statistical analysis

No hypothesis was made for the present study, so sample size estimation was unavailable. Data were expressed as mean ± standard deviation, median [interquartile range], or median (range) for continuous variables and number (%) for categorical variables. Differences between survivors and non-survivors were explored using two-sample *t* test for parametric variables, Wilcoxon rank-sum test for non-parametric variables, and Fisher’s exact test for categorical variables. Kaplan-Meier plot was used for survival data. Age was dichotomized at 65 years. Lymphocyte counts at ICU admission were dichotomized at 1.1 ×  10^9^/L, the lower limit of normal range, and at 0.55 × 10^9^/L and platelet counts at 125 × 10^9^/L. Dichotomized age, lymphocyte counts and platelet counts, and comorbidities and dichotomous complications showing a *p* value < 0.2 in univariate analysis were included for Cox proportional-hazards regression analysis.

All statistical tests were 2-tailed with significance set at *p* value less than 0.05. The Stata/IC 15.1 software (StataCorp, College Station, TX, USA) was applied for all analyses.

## Results

### Demographic data and comorbidities of included patients

**Fig. 1:**
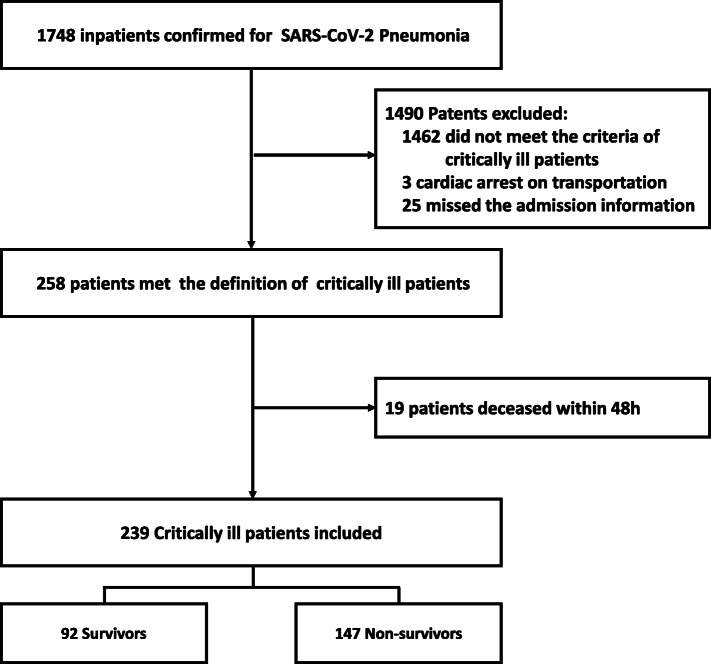
Flowchart of study of the included patients with COVID-2019. COVID-19, coronavirus disease 2019; MV, mechanical ventilation; DNR, do-not-resuscitate

From January 12 to February 3, 2020, a total of 1748 patients with confirmed COVID-19 from the three study centers were screened, and 258 (14.8%) critically ill patients were identified. After excluding 19 patients who deceased within 48 h after ICU admission, 239 patients were included (Fig. 1). The three most common symptoms were fever (218 patients, 91.2%), cough (178 patients, 74.5%), and dyspnea (119 patients, 49.79%) (Supplementary table 1) Their mean age was 62.5 ± 13.3 years, including 112 (46.9%) patients over 65 years old (Table 1). One hundred sixty-two (67.8%) patients had one or more coexisting conditions, including hypertension in 105 (43.9%) patients, chronic cardiac disease in 35 (14.6%) patients, chronic pulmonary disease in 12 (5.0%) patients, cerebrovascular disease in 13 (5.4%) patients, chronic hepatic disease in 20 (8.4%) patients, malignancy in 13 (5.4%) patients, and diabetes mellitus in 44 (18.4%) patients.

**Table 1:** Demographic data and preexisting comorbidities in 239 critically ill patients with COVID-19

Characteristics	All patients (***n*** = 239)	Non-survivors (***n*** = 147)	Survivors (***n*** = 92)	***p*** value, survivors vs non-survivors
Age, mean ± SD, years	62.5 ± 13.3	65.7 ± 12.2	57.5 ± 13.5	< 0.001
Age ≥ 65 years	112 (46.9%)	82 (55.8%)	30 (32.6%)	< 0.001
Male	143 (59.8%)	90 (61.2%)	53 (57.6%)	0.337
APACHE II, median [IQR]^a^	15 [13–17]	15 [13–17]	13 [11–15]	0.006
SOFA score^a^	6 [5–7]	6 [5–7]	5 [5–6]	0.0163
**Preexisting comorbidities**				
Hypertension	105 (43.9%)	64 (43.5%)	41 (44.6%)	0.491
Chronic cardiac disease	35 (14.6%)	21 (14.3%)	14 (15.2%)	0.492
Chronic pulmonary disease	12 (5.0%)	9 (6.1%)	3 (3.3%)	0.253
Cerebrovascular disease	13 (5.4%)	9 (6.1%)	4 (4.3%)	0.392
Chronic liver disease	20 (8.4%)	13 (9.4%)	7 (8.4%)	0.511
Malignancy	13 (5.4%)	11 (7.5%)	2 (2.2%)	0.066
Diabetes	44 (18.4%)	24 (17.7%)	18 (19.6%)	0.420

*COVID-19* coronavirus disease 2019, *SD* standard deviation, *APACHE II* Acute Physiology and Chronic Health Evaluation II, *IQR* interquartile range

Data were expressed as count (%) unless otherwise

^a^APACHE II scores at ICU admission were available in 165 patients, because arterial blood gas analysis was conducted in 101 non-survivors and 64 survivors

### Laboratory tests at ICU admission

The laboratory findings of all critically ill patients on ICU admission were summarized in Table 2. At ICU admission, 219 (91.6%) patients had lymphocyte counts less than 1.1 × 10^9^/L and 103 (43.1%) had lymphocyte counts less than 0.55 × 10^9^/L. A total of 59 (24.7%) patients had platelet count less than 125 × 10^9^/L. Among 165 patients with an analysis of arterial blood gas, their partial pressure of oxygen divided by fraction of inspired oxygen was 91.3 [IQR, 66.6–133.5] mmHg.

**Table 2:** Laboratory tests at ICU admission in 239 critically ill patients with COVID-19

Laboratory tests at ICU admission	All patients (***n*** = 239)	Non-survivors (***n*** = 147)	Survivors (***n*** = 92)	***p*** value, survivors vs non-survivors
Hemoglobin, g/L	124.9 ± 17.9	124.8 ± 17.3	124.9 ± 19.0	0.966
White blood cell count, × 10^9^/L	7.9 [5.2–10.7]	8.2 [5.1–11.0]	7.2 [5.2–10.4]	0.444
Neutrophil count, × 10^9^/L	6.9 [4.0–19.7]	7.2 [4.0–9.7]	6.5 [4.0–9.3]	0.485
Lymphocytes, × 10^9^/L	0.6 [0. 5–0.8]	0.6 [0.4–0.8]	0.7 [0.50–0.9]	0.008
Lymphocyte count < 1.1 × 10^9^/L^**†**^	219 (91.6%)	139 (94.6%)	80 (87.0%)	0.036
Lymphocyte count < 0.55 × 10^9^/L	103 (43.1%)	73 (50.0%)	30 (32.6%)	0.007
Platelets, × 10^9^ /L	175 [125–219]	160 [110–206]	186 [148–232]	< 0.001
Platelet count < 125 × 10^9^ /L^‡^	59 (24.7%)	48 (32.7%)	11 (12.0%)	< 0.001
PT, s	11.9 ± 1.7	12.0 ± 1.8	11.6 ± 1.4	0.079
APTT, s	29.3 ± 9.0	29.3 ± 8.9	29.4 ± 10.9	0.988
Total bilirubin, μmol/L	12.5 [10.3–16.8]	13.0 [10.8–18.7]	11.7 [9.5–15.8]	0.029
ALT, U/L	36 [22–56]	35 [21–54]	39 [26–59]	0.210
AST, U/L	41 [33–62]	45 [35–64]	38 [31–61]	0.051
Serum creatinine, U/L	72.1 [58–85.3]	73.2 [59.7–92.5]	70.2 [54.1–81.8]	0.018
hsTNI, pg/mL	85.5 [35.5–133.5]	91.0 [44.0–134.0]	80.0 [30.0–32.0]	0.410
Myoglobin, ng/mL	105.0 [54.0–155.0]	100.0 [54.0–150.0]	110.5 [56.0–162.5]	0.454
IL-6, pg/mL^‖^	9.1 [6.7–12.0]	9.1 [7.1–12.9]	9.1 [6.2–11.7]	0.385
Elevated IL-6 level^¶^	185 (77.4%)	119 (81.0%)	66 (71.7%)	0.068
Arterial blood gas analysis^*^				
pH	7.46 ± 0.07	7.46 ± 0.08	7.45 ± 0.05	0.496
pO_2_/FiO_2_, mm Hg	91.3 [66.6–133.5]	82.4 [61.6–120.4]	109.0 [77.5–155.6]	0.005
pCO_2_, mmHg	35.1 ± 10.8	35.6 ± 12.9	34.4 ± 6.0	0.477
HCO_3_^−^, mmol/L	24.4 ± 4.0	24.3 ± 4.4	24.5 ± 3.3	0.839

*COVID-19* coronavirus disease 2019, *ICU* intensive care unit, *PT* prothrombin time, *APTT* activated partial thromboplastin time, *ALT* alanine aminotransferase, *AST* aspartate aminotransferase, *hsTNI* hypersensitive troponin I, *IL-6* interleukin 6, *pO*_*2*_ partial pressure of oxygen, *FiO*_*2*_ fraction of inspired oxygen, *pCO*_*2*_ partial pressure of carbon dioxide

Data were expressed median [interquartile range] or as mean ± standard deviation

^†^The lower limit of normal range of lymphocyte count was 1.1 × 10^9^/L

^‡^The lower limit of normal range of platelet count was 125 × 10^9^/L

^‖^IL-6 analysis was conducted in 114 non-survivors and 67 survivors

^¶^The upper limit of normal range was 7 pg/ml

*Arterial blood gas analysis was conducted in 101 non-survivors and 64 survivors

### Complications, treatments, and medications

**Fig. 2:**
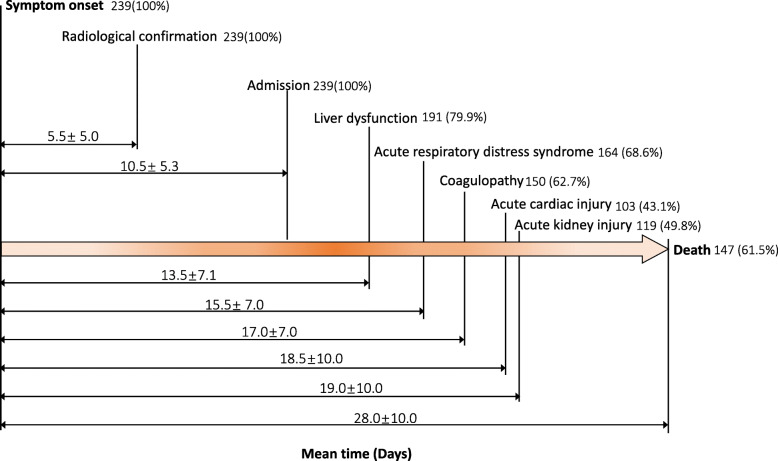
Clinical course of critically ill patients with COVID-19. COVID-19, coronavirus disease 2019; ARDS, acute respiratory distress syndrome AKI, acute kidney injury

Complications of 239 critically ill patients included ARDS in 164 (68.6%) patients, acute cardiac injury in 103 (43.1%) patients, liver dysfunction in 191 (79.9%) patients, AKI in 119 (49.8%) patients, coagulopathy in 150 (62.7%) patients, bacterial pneumonia in 25 (10.5%) patients, and bacteremia 10 (4.2%) patients (Table 3). Chronologically, liver dysfunction, ARDS, coagulopathy, acute cardiac injury, and AKI occurred 13.5 days, 15.5 days, 17 days, 18.5 days, and 19 days after the symptom onset, respectively (Fig. 2).

**Table 3:** Complications, treatments, and medications in 239 critically ill patients with COVID-19

Characteristics	All patients (***n*** = 239)	Non-survivors (***n*** = 147)	Survivors (***n*** = 92)	***p*** value, survivors vs non-survivors
**Complications**				
ARDS	164 (68.6%)	118 (80.3%)	46 (50.0%)	< 0.001
Acute cardiac injury	103 (43.1%)	81 (55.1%)	22 (23.9%)	< 0.001
AKI	119 (49.8%)	99 (67.4%)	20 (21.7%)	< 0.001
Liver dysfunction	191 (79.9%)	127 (86.4%)	64 (69.6%)	< 0.001
Coagulopathy	150 (62.7%)	111 (75.5%)	39 (42.4%)	< 0.001
Hospital-acquired infection				
Bacterial pneumonia	25 (10.5%)	16 (10.9%)	9 (9.8%)	0.484
Bacteremia	10 (4.2%)	8 (5.4%)	2 (2.2%)	0.187
Urinary tract infection	5 (2.1%)	4 (2.7%)	1 (1.1%)	0.652
**Treatments**				
Mechanical ventilation	165 (69.0%)	119 (81.0%)	46 (50.0)	< 0.001
Invasive	79 (33.1%)	71 (48.3%)	8 (8.7%)	< 0.001
Noninvasive	136 (56.1%)	95 (64.6%)	41 (43.6%)	0.002
Noninvasive + invasive	50 (20.9%)	47 (32.0%)	3 (3.3%)	< 0.001
Extracorporeal membrane oxygenation	9 (3.8%)	9 (6.1%)	0 (0.0%)	0.011
Renal replacement therapy	12 (5.0%)	11 (7.5%)	1 (1.1%)	0.022
**Medications**				
Antiviral agents	132 (55.2%)	82 (55.8%)	50 (54.5%)	0.466
Arbidol	77 (32.2%)	46 (31.3%)	31 (33.7%)	0.776
Interferon alpha	91 (38.1%)	56 (38.1%)	35 (38.0%)	1.000
Oseltamivir	59 (24.7%)	32 (21.8%)	27 (29.4%)	0.218
Lopinavir/ritonavir	38 (15.9%)	24 (16.3%)	14 (15.2%)	0.858
Ribavirin	13 (5.4%)	6 (4.1%)	7 (7.6%)	0.255
Ganciclovir	41 (17.2%)	21 (14.3%)	20 (21.7%)	0.159
Antibacterial agents	229 (95.8%)	144 (98.0%)	85 (92.4%)	0.041
Methylprednisolon	189 (79.1%)	118 (80.3%)	71 (77.2%)	0.339
Commutative dosages, median [IQR], mg	360 [200–580]	370 [160–640]	320 [240–580]	0.517
Dose, methylprednisolon equivalent/days, mg	60.9 ± 21.7	64.6 ± 23.0	54.7 ± 17.9	0.0021
Duration of methylprednisolon, days	6 [4–10]	6 [3–10]	7 [5–11]	0.0345
Immunoglobulin	138 (57.7%)	94 (64.0%)	44 (47.8%)	0.010
Thymosin α1	103 (43.10)	67 (45.6%)	36 (39.1%)	0.199
Length of ICU stay, median [IQR], days	17 [10–26]	12 [8–18]	26.5 [19–46.5]	< 0.001

*ARDS* acute respiratory distress syndrome, *AKI* acute kidney injury, *IQR* interquartile range

Data were expressed as count (%) unless otherwise

**Fig. 3:**
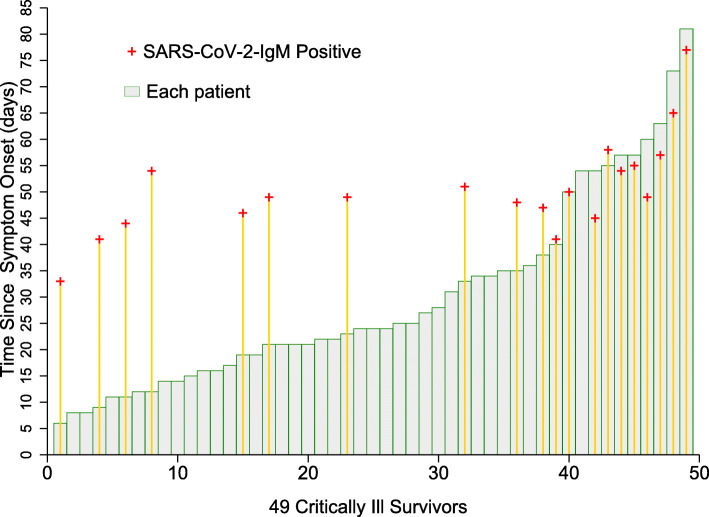
Durations of negative conversion of SARS-CoV-2 RNA in 49 critically ill survivors. Each bar indicates one survivor and the height of bars indicates duration between symptom onset and the day of last positive test for SARS-CoV-2 RNA. The red plus markers indicate the last positive tests of SARS-CoV-2 IgM. SARS-CoV-2, severe acute respiratory coronavirus 2; COVID-19, coronavirus disease 2019

In 49 patients with data on negative conversion of SARS-CoV-2 RNA, the median duration since symptom onset was 30 (range 6–81) days. Among these patients, 20 (40.8%) patients tested positive for SARS-CoV-2 IgM (Fig. 3).

One hundred and sixty-five (69.0%) patients received mechanical ventilation (MV). Of these patients, 29 (17.6%) patients only received invasive ventilation (IMV), 86 (52.1%) patients only received noninvasive ventilation (NIV), and the remaining 50 (30.3%) patients received both, including only 1 patient receiving NIV after removal of endotracheal tube.

Of all patients, 9 (3.8%) patients with refractory hypoxemia received salvage therapy of extracorporeal membrane oxygenation (ECMO), while 12 (5.0%) patients received renal replacement therapy.

Antimicrobial and antiviral agents were given to 229 (95.8%) and 132 (55.2%) patients, respectively. The different antiviral therapy was summarized in Table 3. Methylprednisolone, the only type of glucocorticoids used, was given to 189 (79.1%) patients, with a median commutative dosage of 360 [IQR, 200–560] mg, and the mean daily dose of methylprednisolone was 60.9 ± 21.7 mg.

### Mortality at 60 days and its predictors

**Fig. 4:**
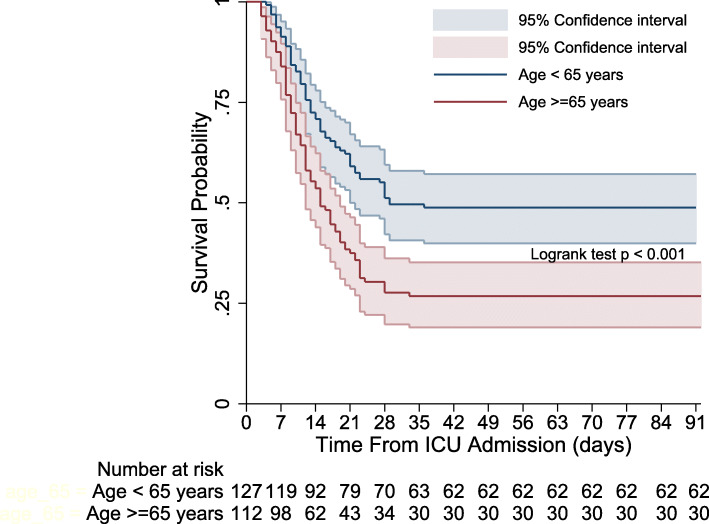
Survival curves of 239 critically ill patients with COVID-19. The number of patients ≥ 65 years and patients < 65 years was 112 and 127, respectively. COVID-19, coronavirus disease 2019

One hundred and forty-seven (61.5%) patients deceased by 60 days after ICU admission. Twenty-eight patients deceased before receiving MV, comprising 2 patients with do-not-resuscitate, and 26 patients due to extra-respiratory causes. For 112 patients aged 65 years or older, 82 (73.2%) deceased, and for 127 patients under 65 years old, 65 (51.2%) deceased (Fig. 4). The mean duration between symptom onset and decease was 24.5 days (Fig. 2). The median duration between ICU admission and decease was 12 (range 3–36).

Of all critically ill patients, by April 5th, 2020, 85 (35.6%) patients had been discharged, including 5 patients on oxygen therapy using common nasal cannula at home. Seven patients were still being hospitalized, and their oxygen therapy comprised common nasal cannula in 5 patients, high-flow nasal cannula in 1 patient, and invasive ventilator in 1 patient.

In univariate analysis, in comparison with survivors, non-survivors were older (65.7 ± 12.2 years vs 57.5 ± 13.5 years, *p* <  0.001) with more patients older than 65 years (55.8% vs 32.6%, *p* <  0.001) (Table 1), higher APACHE II scores (15 [IQR, 13–17] in 90 patients vs 13 [IQR, 11–15] in 74 patients, *p* = 0.006) (Table 1), higher SOFA scores (5 [IQR, 5–7] in 90 patients vs 5 [IQR, 5–6] in 74 patients, *p* = 0.0163) (Table 1), less counts of lymphocytes (0.6 [IQR, 0.4–0.8] × 10^9^/L vs 0.67 [IQR, 0.5–0.9] × 10^9^/L, *p* = 0.008) with more patients with lymphocyte count less than 1.1 × 10^9^/L (94.6% vs 87.0%, *p* = 0.036) and more with lymphocyte count less than 0.55 × 10^9^/L (50.0% vs 32.6%, *p* = 0.007), less counts of platelets (160 [IQR, 110–206] vs 186 [IQR, 148–232], *p* <  0.001) with more patients with platelet count less than 125 × 10^9^/L (32.7% vs 12.0%, p <  0.001). Compared with survivors, more non-survivors developed ARDS (80.3% % vs 50.0%, *p* <  0.001), acute cardiac injury (55.1% vs 23.9%, *p* <  0.001), AKI (67.4% vs 21.7%, *p* <  0.001), liver dysfunction (86.4% vs 69.6%, *p* <  0.001), and coagulopathy (75.5% vs 42.4%, *p* <  0.001). No significant difference between the two groups was identified in pharmacological therapies, except immunoglobulin (94 (64.0%) in survivors vs 44 (47.8%) in non-survivors, *p* = 0.010) (Table 3). Survivors had significantly longer length of ICU stay than non-survivors (26.5 [19–46.5] days vs 12 [8–18] days, *p* <  0.001) (Table 3).

The unviable analysis result (columns 2–4 in Table 4) shows the risk effect in survival time solely determined by a predictor, without considering its relation with other risk factors. The multivariable Cox model result can be interpreted, and when multiple risk factors present simultaneously, some factors may be able to delegate the other factors’ risk effects. Cox proportional-hazards regression analysis stratified by study center revealed that age older than 65 years (hazards ratio (HR), 1.57 (95% CI 1.12–2.19), *P* = 0.009), platelet count less than 125 × 10^9^/L at ICU admission (HR 2.01, (95%CI, 1.39–2.91), p <  0.001), ARDS (HR 1.67 (95%CI, 1.05–2.64), *P* = 0.029), and AKI (HR 2.06, (CI, 1.36 to 3.10), *p* = 0.001) were independent predictors of 60-day mortality (Table 4).

**Table 4:** Predictors of 60-day mortality in 239 critically ill patients with COVID-19 identified using Cox proportional-hazards model

Characteristics	Non-survivors (***n*** = 147)	Survivors (***n*** = 92)	***p*** value	Cox proportional-hazards model	
				Hazard ratio (95% confidence interval)	*p* value
Age ≥ 65	82 (55.8%)	30 (32.6%)	< 0.001	1.57 (1.12–2.19)	0.009
Malignancy	11 (7.5%)	2 (2.2%)	0.066	1.62 (0.84–3.14)	0.149
Lymphocyte count < 0.55 × 10^9^/L^**†**^	73 (50.0%)	30 (32.6%)	0.007	1.23 (0.88–1.73)	0.226
Platelet count < 125 × 10^9^ /L^‡^	48 (32.7%)	11 (12.0%)	< 0.001	2.01 (1.39–2.91)	< 0.001
ARDS	118 (80.3%)	46 (50.0%)	< 0.001	1.67 (1.05–2.64)	0.029
Acute cardiac injury	81 (55.1%)	22 (23.9%)	< 0.001	0.88 (0.57–1.34)	0.542
AKI	99 (67.4%)	20 (21.7%)	< 0.001	2.06 (1.36–3.10)	0.001
Liver dysfunction	127 (86.4%)	64 (69.6%)	< 0.001	1.40 (0.77–2.55)	0.264
Coagulopathy	111 (75.5%)	39 (42.4%)	< 0.001	1.40 (0.88–2.21)	0.156

*ARDS* acute respiratory distress syndrome, *AKI* acute kidney injury

Data were expressed as count (%) unless otherwise

^†^The low limit of normal range of lymphocyte count was 1.1 × 10^9^/L.

^‡^The low limit of normal range of platelet count was 125 × 10^9^/L

## Discussion

In this multicenter retrospective study on critically ill patients with COVID-19, the main findings include that the critically ill patients requiring ICU admission and mechanical ventilation or oxygen therapy with FiO_2_ greater than or equal to 60% had a considerable 60-day mortality and that age older than 65 years, thrombocytopenia at ICU admission, ARDS, and AKI were independent predictors of 60-day mortality of these patients.

After excluding all the patients included in the previous study and incorporating two more centers, the mortality we found in the present study was similar to our previous study [2]. Compared to the present study, the previous study was a preliminary casualty report with a smaller sample size. On February 17th, the Chinese CDC stated that of 2087 critically ill patients with COVID-19, the case fatality rate was 49.0% [18]. The study was somewhat cross-sectional. It is impossible that 51.0% of patients in the denominator had been treated more than 60 days or discharged alive, so it is reasonable that the mortality rate of critically ill patients should be higher than 49.0%. According to the official guidelines, critically ill patients were those who need mechanical ventilator support, are in shock, or need supports for other dysfunctional organs [18]. The mortality of critically ill patients with COVID-19 reported in studies outside China was also high. In the first series of critically ill patients with COVID-19 in Washington, USA, the mortality was 67% [19]. In a study of 1591 critically ill patients from Lombardy Region, Italy, 26% deceased and 58% were still in ICU [20]. In another study, 282 (88.1%) of 320 patients who received mechanical ventilation deceased [21].

Advanced age, ARDS, and AKI influenced the mortality of critically ill patients with COVID-19 in an intertwined way. There were debates on whether COVID-19 caused typical ARDS, mainly because some patients were with preserved lung compliance [22]. But a recent postmortem study showed that the fundamental pathological characteristics of pulmonary infection caused by SARS-CoV-2 in critically ill patients were diffuse alveolar damage, which was also the typical pathological finding of severe acute respiratory syndrome and Middle East respiratory syndrome [23]. We believe alveolar damage occurs at early the stage of severe COVID-19, and some of these patients recovered gradually, some deceased in a short time, and the rest deteriorated with alveolar damage progressing, fulfilling the diagnostic criteria of ARDS at some point. Like in critically ill patients with SARS and MERS [24–26], older critically ill patients with COVID-19 had a higher mortality [2]. In older patients, both the incidence and mortality of ARDS were higher [27–30]. Both mild-moderate and severe ARDS are associated with a substantial increase in mortality in patients with AKI [31].

Besides the general association among advanced age, ARDS, and AKI, SARS-CoV-2 induces complications by binding to the angiotensin-converting enzyme 2 (ACE2) receptor [5, 32]. Angiotensin-converting enzyme 2 (ACE2), the same cell entry receptor for both SARS-CoV and SARS-CoV-2, plays an important role in organ damages in patients infected by either virus [32–34]. The primary target organ of SARS-CoV is the lung [35]. In the respiratory tract, angiotensin-converting enzyme 2 (ACE2) is widely expressed on the epithelial cells and macrophages of alveoli, which facilitates the progression of ARDS, and thereafter causes death [3, 34, 36]. As a surface molecule, ACE2 also diffusely locates on epithelial cells of the renal tubules [37]. SARS-CoV particles have been detected in the cytoplasm of these cells in postmortem studies, which explains negative conversion of SARS-CoV in urine [38]. Recently, negative conversion of SARS-CoV-2 RNA in urine has also been confirmed [4]. The destruction of epithelial cells in the renal tubules leads to acute tubular necrosis, the most common form of AKI [37]. In a previous study, advanced age and ARDS are identified as independent risk factors for development of AKI, and AKI was known as an important indicator for death in patients with SARS [39].

The mechanisms of thrombocytopenia in patients with COVID-19 are not clear right now [40]. Mechanisms of SARS-CoV-2-induced thrombocytopenia have been suggested [41, 42]. On one hand, SARS-CoV-2 infects bone marrow cells, thereby causing abnormal hematopoiesis. On the other hand, the lung tissue damages with SARS-CoV-2 infection also cause platelet aggregation and consumption of platelets. The latter has been demonstrated by the findings of hemorrhagic necrosis in pathological examination of lungs from critically ill patients with COVID-19 [43].

In the process of diagnosing and treating patients with COVID, qualitative evaluations (positive or negative) of samples from the respiratory tract are widely used [44, 45]. In some studies, quantitative evaluations are mainly used in exploratory studies, showing that a high load of SARS-CoV-2 may be an indicator of the severity of infection [46, 47]. A median (range) duration of 30 (6–81) days between symptom onset and negative conversion of SARS-CoV-2 RNA in some critically ill survivors is surprising. The inability to clear off the virus in a short time may lead to a prolonged period of critical condition in these patients. But whether they can still spread the virus needs further study [48]. Studies on antibody responses against SARS-CoV-2 are also needed.

Our previous study showed that compared with survivors, non-survivors were more likely to have preexisting comorbidities, which were not identified in the present study, except malignancy in univariate analysis [2]. The major reason was the exclusion of patients who deceased within 48 h after ICU admission. This is one limitation of the present study. In a study of patients with COVID-19 including 137 survivors and 54 non-survivors, Zhou et al. conducted a multivariable logistic regression analysis to identify risk factors of death without including severe complications after hospital admission [7]. We conducted the present study to explore the treating effect of critical care deeply by excluding the dying patients who deceased in 48 h after ICU admission. Different predicting effects of preexisting comorbidities will be identified due to different study designs [49]. The second limitation was that some important information was not available, especially PO_2_/FiO_2,_ which caused a failure to include APACH II score into the Cox proportional-hazards regression model. Third, 80% of included patients were from Jin-yintan Hospital, and all included patients from Jin-yintan Hospital were transferred from other hospitals. The details of treatment before admission to Jin-yintan Hospital were unavailable. But treating patients in designated hospitals was one paramount measure in dealing with the COVID-19 epidemic [50]. And we conducted Cox proportional-hazards regression stratified by study centers. Fourth, the sample size is not large enough, and other variables remain to be explored. We are expecting further studies.

## Conclusion

The mortality of critically ill patients with COVID-19 is considerably high and age older than 65 years, thrombocytopenia at ICU admission, ARDS, and AKI are independent predictors of 60-day mortality. The duration between symptom onset and negative conversion of SARS-CoV-2 RNA and its association with the severity of critically ill patients should be seriously considered in treating patients with COVID-19 and needs to be studied.

## Supplementary information

**Additional file 1: S1.** Symptoms of 239 critically ill patients with COVID-19.

## Data Availability

After publication, data are available upon reasonable request. A proposal with a detailed description of study objectives and statistical analysis plan will be needed for evaluation of the reasonability of requests. Additional materials might also be required during the process of evaluation. Deidentified participant data will be provided after approval from the corresponding author and Wuhan Jin Yin-tan Hospital.

## Abbreviations

AKI: Acute kidney injury
ALT: Alanine aminotransferase
APACHE II: Acute Physiology and Chronic Health Evaluation II
APTT: Activated partial thromboplastin time
ARDS: Acute respiratory distress syndrome
AST: Aspartate aminotransferase
COVID-19: Novel coronavirus disease 2019
ICUs: Intensive care units
ECMO: Extracorporeal membrane oxygenation
FiO_2_: Fraction of inspired oxygen
hsTNI: Hypersensitive troponin I
IQR: Interquartile range
IL-6: Interleukin 6
pO_2_: Partial pressure of oxygen
pCO_2_: Partial pressure of carbon dioxide
PT: Prothrombin time
SARS-CoV-2: Severe acute respiratory syndrome coronavirus 2
