# Supplementary material for: Clinical course and predictors of 60-day mortality in 239 critically ill patients with COVID-19: a multicenter retrospective study from Wuhan, China
Source: Crit Care. 2020 Jul 6;24:394. doi: 10.1186/s13054-020-03098-9 (PMC7336107; doi:10.1186/s13054-020-03098-9)
Supplement: Supplementary file 1 — Additional file 1: S1. Symptoms of 239 critically ill patients with COVID-19. [file 13054_2020_3098_MOESM1_ESM.pdf]

S1. Symptoms of 239 critically ill patients with COVID-19

**Symptoms**	**All patients (n=239)**
Fever	218 (91.2%)
Cough	178 (74.5%)
Dyspnea	119 (49.79%)
Sore throat	11 (4.6%)
Myalgia	20 (8.47%)
Malaise	68 (28.5 %)
Rhinorrhea	3 (1.3 %)
Arthralgia	7 (3.0%)
Chest pain	9 (3.8%)
Headache	8 (3.4%)
Vomiting	6 (2.9%)
Diarrhea	8 (3.4%)

Data were expressed as count (%).
